# Comparison of laparoscopy-assisted and open radical gastrectomy for advanced gastric cancer: A retrospective study in a single minimally invasive surgery center

**DOI:** 10.1097/MD.0000000000003936

**Published:** 2016-06-24

**Authors:** Yingxue Hao, Peiwu Yu, Feng Qian, Yongliang Zhao, Yan Shi, Bo Tang, Dongzhu Zeng, Chao Zhang

**Affiliations:** Department of General Surgery, Center for Minimally Invasive Gastrointestinal Surgery, Southwest Hospital, Third Military Medical University, Chongqing, China.

**Keywords:** advanced, gastrectomy, laparoscopy, radical dissection, stomach neoplasms, surgery

## Abstract

Laparoscopy-assisted gastrectomy (LAG) has gained international acceptance for the treatment of early gastric cancer (EGC). However, the use of laparoscopic surgery in the management of advanced gastric cancer (AGC) has not attained widespread acceptance. This retrospective large-scale patient study in a single center for minimally invasive surgery assessed the feasibility and safety of LAG for T2 and T3 stage AGC. A total of 628 patients underwent LAG and 579 patients underwent open gastrectomy (OG) from Jan 2004 to Dec 2011. All cases underwent radical lymph node (LN) dissection from D1 to D2+. This study compared short- and long-term results between the 2 groups after stratifying by pTNM stages, including the mean operation time, volume of blood loss, number of harvested LNs, average days of postoperative hospital stay, mean gastrointestinal function recovery time, intra- and post-operative complications, recurrence rate, recurrence site, and 5-year survival curve. Thirty-five patients (5.57%) converted to open procedures in the LAG group. There were no significant differences in retrieved LN number (30.4 ± 13.4 vs 28.1 ± 17.2, *P* = 0.43), proximal resection margin (PRM) (6.15 ± 1.63 vs 6.09 ± 1.91, *P* = 0.56), or distal resection margin (DRM) (5.46 ± 1.74 vs 5.40 ± 1.95, *P* = 0.57) between the LAG and OG groups, respectively. The mean volume of blood loss (154.5 ± 102.6 vs 311.2 ± 118.9 mL, *P* < 0.001), mean postoperative hospital stay (7.6 ± 2.5 vs 10.7 ± 3.6 days, *P* < 0.001), mean time for gastrointestinal function recovery (3.3 ± 1.4 vs 3.9 ± 1.5 days, *P* < 0.001), and postoperative complications rate (6.4% vs 10.5%, *P* = 0.01) were clearly lower in the LAG group compared to the OG group. However, the recurrence pattern and site were not different between the 2 groups, even they were stratified by the TNM stage. The 5-year overall survival (OS) rates were 85.38%, 79.70%, 57.81%, 34.60% and 88.31%, 75.49%, 56.84%, 33.08% in patients with stage Ib, IIa, IIb, and IIIa, respectively, in the LAG and OG groups. There were no statistically significant differences in the OS rate for patients with the same TNM stage between the 2 groups. LAG with radical LN dissection is a safe and technically feasible procedure for the treatment of AGC staged below T3.

## 1 Introduction

Kitano performed the first report of laparoscopy-assisted distal gastrectomy (LADG) in 1994,^[1]^ and laparoscopy-assisted gastrectomy (LAG) has gained acceptance as one of the best treatments for early gastric cancer (EGC).^[2]^ Therefore, the number of patients undergoing LAG is increasing. Several recent randomized controlled trails (RCTs) demonstrated that LAG was technically feasible for peri-gastric lymph node (LN) dissection for EGC. These studies reported very low surgical morbidity and mortality rates and improvements in postoperative quality of life that were comparable with conventional open surgery.^[3–6]^

However, LAG for advanced gastric cancer (AGC) has not attained widespread acceptance, and it remains limited to several medical centers.^[7–10]^ The reasons of this slow acceptance are as the following major concerns: (1) if can achieve free tumor margins during laparoscopic procedure; (2) if can dissect sufficient LNs; and (3) the possibility that the laparoscopic procedure promotes cancer cells dissemination.^[11–13]^ Some authors recently reported that LAG is safe for AGC; this technique can achieve tumor-free margins and a higher mean number of retrieved LNs.^[14–16]^ However, the feasibility and safety of LAG for AGC lacks large-scale and RCT study data. The present study was a relatively large-scale case study (628 LAG and 579 open gastrectomies [OG]) at a single minimally invasive surgery center. We compared the clinicopathological characteristics of patients, surgical procedures, and short- and long-term outcomes of laparoscopy-assisted and open radical gastrectomy for AGC.

## 2 Methods

### 2.1 Patients

The present study included 628 patients with AGC who underwent LAG and 579 patients who underwent OG in a minimally invasive surgery center from January 2004 through December 2011. The study was performed in accordance with the Declaration of Helsinki (2000) of the World Medical Association. The Ethics Committee of Southwest Hospital approved this study. Informed consent was obtained from each patient preoperatively after a detailed explanation of the LAG and OG procedures was provided. All patients agreed to participate in this study. We used histological examination during endoscopic examination to make a definite diagnosis of gastric cancer. Tumor site and invasive degree were confirmed by the endoscopic, barium, and endoscopic ultrasonographic (EUS) findings. The patients were all examined by chest, abdomen, and pelvis computed tomography (CT) to find the LN and distant metastases. The inclusion criteria for this study were as follows: curative dissection, pathologically confirmed gastric adenocarcinoma, an R0 resection, no evidence of distant metastases, and no adjuvant chemoradiotherapy prior to surgery. Patients who demonstrated clinical evidence of distant metastases, with T1 stage or greater than T4 stage, or a history of previous malignant disorders or gastrectomy for benign and malignant disease were excluded.

All patients in the 2 groups underwent partial or total gastrectomy with D1 to D2+ lymphadenectomy according to the Japanese Gastric Cancer Treatment Guidelines (ver. 3).^[17]^ Patients in both groups received 6 cycles of chemotherapy after operation with cisplatin and 5-fluorouracil. Follow-up were performed by telephone calls, medical records, and mail. All the patients were followed up using blood tests, tumor markers, chest radiography, CT scans of the abdomen and pelvis, and alternating endoscopy or positron emission tomography (PET). Follow-up studies were performed at ∼3-month intervals in the first year, and patients were followed at 6-month intervals for the next 2 years, and annually thereafter.

### 2.2 Surgical procedure

Five surgical teams experienced in laparoscopic and open gastrectomy techniques performed all surgeries. The patients chose the surgical procedure (open vs laparoscopic) by their individual decision after they were informed of the methods and risks of each procedure. In the early period, most of them choose the open approach because they did not understand the minimally invasive approach. However, the ration of laparoscopic approach was increasing over time after they knew the advantages of laparoscopic surgeries. The laparoscopy-assisted radical gastrectomy procedure was similar to previously reported procedures.^[9,18]^ Briefly, all patients were placed in a supine position under general anesthesia with their legs separated. The initial port was inserted through a 12-mm infra-umbilical incision that was created using the open method. A pneumoperitoneum was established using carbon dioxide (CO_2_) insufflations at a pressure of ∼12 mm Hg. A laparoscope was introduced through the umbilical port. Other trocars were introduced under laparoscopic guidance. The surgeon stood at the patient's left side, the first assistant stood at the right side and the camera assistant was between the patient's legs. Routine exploration of the tumor size and site, the degree of serosa invasion, the peritoneum, and the surface of the liver and other organs (especially ovaries for female patients) were performed prior to beginning the resection. Peritoneal fluid cytology was also obtained. If the results were positive for cancer cells in biopsies of distal tumor deposits or the collected fluid samples, then these patients were identified as stage IV gastric cancer and excluded from the 2 groups.

**Figure 1:**
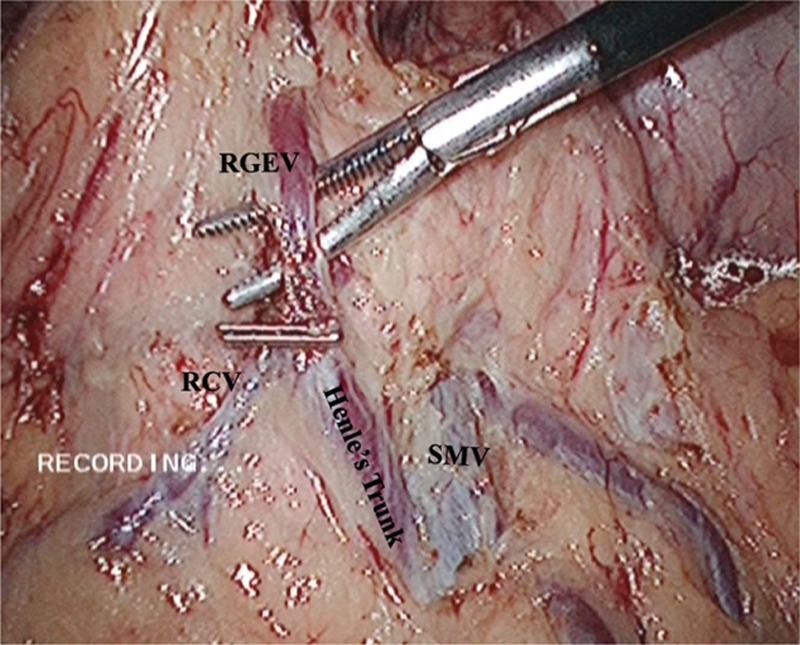
The soft tissues containing LNs no. 6 were removed to reveal the bordering vessels, the right gastroepiploic vein (RGEV), right colic vein (RCV), and Henle's trunk. The area of the no. 14 v LNs was also dissected with the superior mesenteric vein (SMV) exposed. RCV = right colic vein, RGEV = right gastroepiploic vein, SMV = superior mesenteric vein.

**Figure 2:**
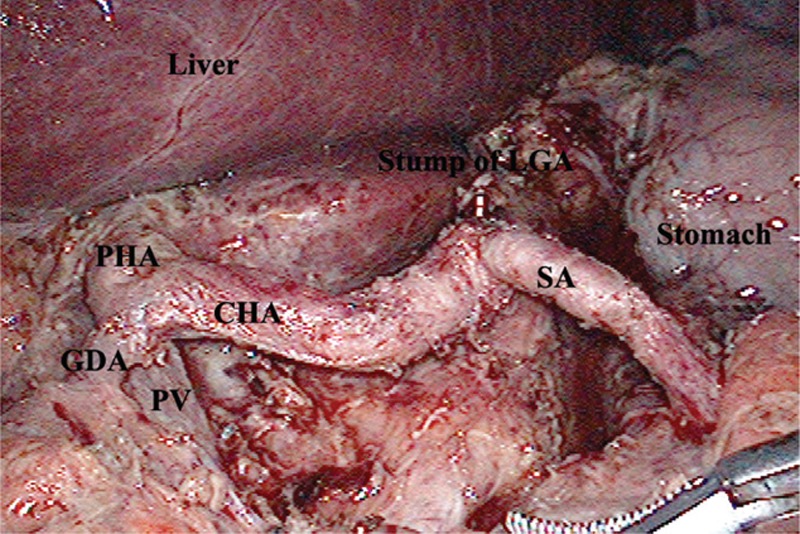
The soft tissues along the celiac axis were cleared to identify the root of the left gastric artery (LGA) and retrieve LNs from stations 7 and 9. The dissection continued along the splenic artery (SA) and common hepatic artery (CHA) to retrieve LNs no. 11p and 8a. CHA = common hepatic artery, LGA = left gastric artery, SA = splenic artery.

For the LADG and D2 LN dissection, first we divided the greater omentum from the transverse colon toward the spleen's lower pole using a Harmonic ACE (Ethicon Endo-Surgery, Cincinnati, OH), and then exposed the left gastroepiploic artery and vein near the tail of the pancreas and cleared the no. 4sb LNs. The second dissection region was inferior to the pylorus. Continuing to divide the greater omentum rightward to the hepatic flexure, the dissecting plane was maintained along the middle colic artery. The superior mesenteric vein, right colic vein, Henle's trunk, and right gastroepiploic vein were exposed, and then the LNs no. 14 v was dissected (Fig. 1). The right gastroepiploic artery was skeletonized, and divided at its origin. The no. 4d and no. 6 LNs were cleared. The third dissection region was superior to the pancreas, which was the most important dissection region. The proximal splenic artery was exposed and the no. 11p LNs were cleared. Then continued to clear the celiac trunk, the left gastric artery, and the common hepatic artery (nos. 9, 7, and 8a) LNs (Fig. 2). Exposed the right gastric artery and proper hepatic artery along the gastroduodenal artery. Cut the right gastric artery, then cleared the no. 5 and no.12a LNs. Finally, cleared the no. 1 and no. 3 LNs along the lesser curvature and the right of esophagocardial junction.

A median superior abdominal incision was performed at ∼4 to 6 cm (according to the size of the tumor), and a wound protector was placed. The stomach was removed and divided using a 90-mm TA stapling device (Ethicon Endo-Surgery, Inc., Cincinnati, OH). The specimen was removed and grossly inspected. Frozen sections were created as needed of the proximal or distal margins. Billroth I gastroduodenostomy was performed using a circular stapler (Proximate CDH 25; Ethicon Endo-Surgery, Inc., Cincinnati, OH), and Billroth II gastrojejunostomy was performed using flexible laparoscopic stapling devices (Echelon Flex, Ethicon Endo-Surgery, Inc., Cincinnati, OH).

For laparoscopy-assisted total gastrectomy (LATG), the dissection was continued upward along the spleen vessels and cleared the LNs of the splenic hilum (no. 10) and the distal splenic artery (no. 11d). Then the LNs along the short gastric vessels (no. 4sa) and around the left paracardial LNs (no. 2) were dissected. Roux-en-Y anastomosis was used for the reconstruction of total gastrectomy. In brief, after completing jejunojejunostomy, we used a circular stapler (Proximate CDH 25; Ethicon Endo-Surgery, Inc., Cincinnati, OH) to perform esophagojejunostomy. For laparoscopy-assisted proximal gastrectomy (LAPG), the right gastroepiploic vessels and right gastric vessels were not divided, and the dissection of the perigastric LNs was performed as described for LATG. Reconstruction of the alimentary tract used esophagogastrostomy, and pyloroplasty was performed in most patients.

For the OG group, a 20- to 25-cm incision was created from the falciform process to the periumbilical area. The same 5 experienced surgical teams performed distal, proximal, or total gastrectomies with radical LN dissection.

### 2.3 Clinical analysis

The following data were attained from the patients’ medical charts: age, gender, history of abdominal surgery, tumor location, histological grade, volume of blood loss, operation time, extent of lymphadenectomy, days of the tumor to the resection margin, days to first flatus, liquid intake and ground activities, length of postoperative hospital stay, intra- and post-operative complications, overall survival (OS) rates, recurrence site and rates, and clinical stage according to the American Joint Committee on Cancer (AJCC) staging criteria (ver. 7).^[19]^ The last date of follow-up was Dec 31, 2012.

### 2.4 Statistical analysis

All the continuous variables are presented as means ± SD (standard deviation), and differences in these variables were analyzed using unpaired 2-group *t* tests. Differences in categorical variables, such as postoperative complications, recurrence rates, and other clinicopathological factors, were analyzed using the chi-square test. Statistical significance was assumed for *P* values < 0.05. The Kaplan–Meier method and log-rank test were used to calculate OS rates and analyze survival differences. SPSS (ver. 18.0) (SPSS, Chicago, IL) was used for all analyses.

## 3 Results

### 3.1 Clinicopathological characteristics

A total of 628 LAG and 579 OG with radical LN dissections were performed for AGC. There was no difference in gender distribution or mean age between the 2 groups. Associated medical illnesses and surgical risks were estimated according to the American Society of Anesthesiologists (ASA) status, and there was no difference between the 2 groups. Most tumors occurred in the lower part of the stomach, rather than the middle or upper part, in both groups. The distribution of malignancy stages ranged from Ib to IIIa, with T3 stage exceeding T2 in both groups. There were no obvious differences in histological grade between the 628 LAG and 579 OG patients. There were 54 (8.6%) patients with abdominal surgical history in the LAG group and 43 (7.4%) in the OG group. The most common prior abdominal surgeries were gynecological surgeries, colectomy and appendectomy. Table 1 shows the clinicopathological characteristics of the study patients.

**Table 1:**
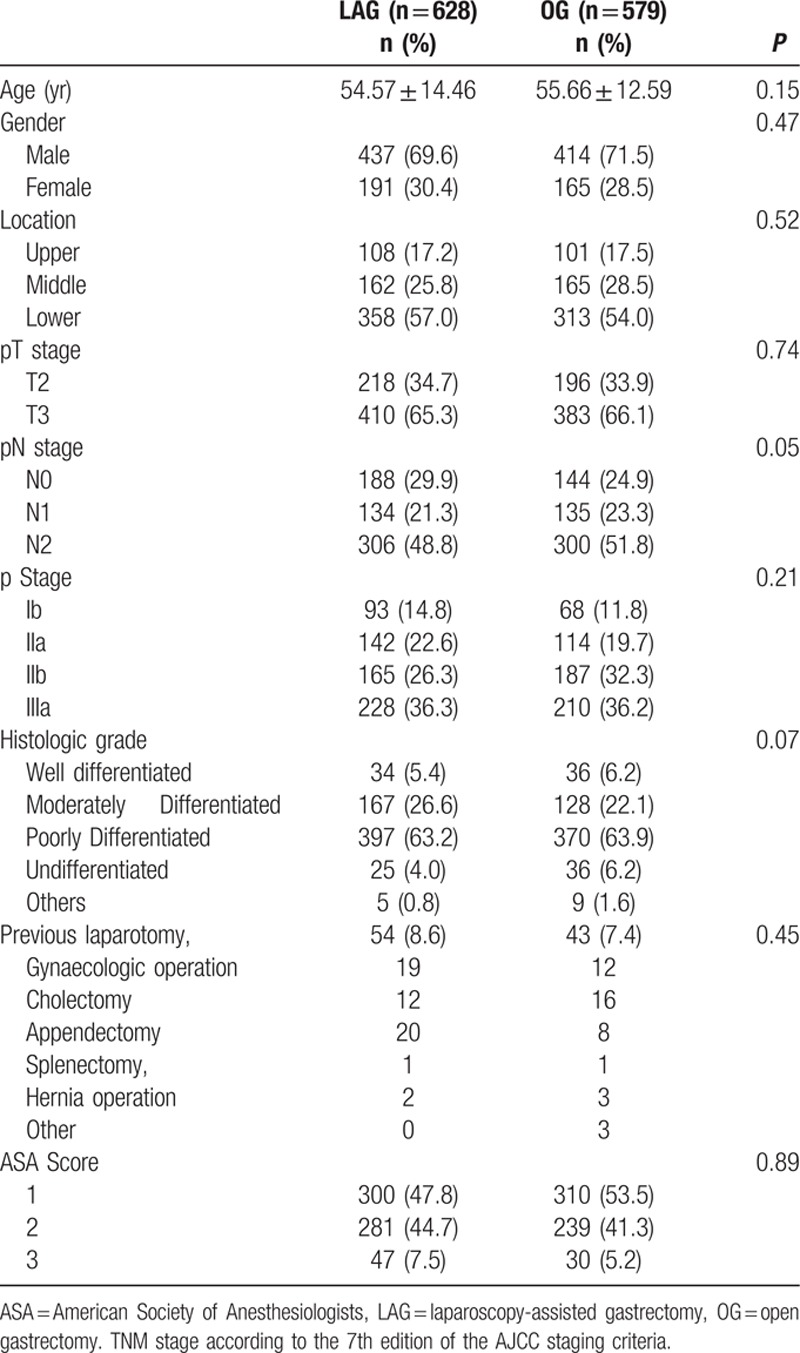
Clinicopathologic characteristics in the LAG and OG groups.

### 3.2 Short-term results

Table 2 shows the procedures performed using laparoscopic and open approaches. Distal subtotal gastrectomy was performed more often than proximal and total gastrectomy in both groups. All resected margins in the LAG and OG groups were free of tumor. There was no significant difference in the extent of lymphadenectomy between the LAG and OG groups (*P* = 0.11). D2 dissection was the most commonly performed dissection in the 2 groups, with 532 (86.3%) and 509 (87.9%) patients in the LAG and OG groups, respectively. The types of alimentary tract reconstruction included Billroth I, Billroth II, Billroth II + Braun, Roux-en-Y, and esophagogastrostomy. Thirty-five cases in the LAG group (5.57% of the total 628 LAG patients) who began laparoscopic surgery were converted to an open procedure: 20 cases occurred during the early 3 years (2004–2006) of performing LAG, which accounted for 11.9% of the total 167 cases performed, and 15 cases were converted during the last 5 years, which accounted for only 3.2% of the 461 patients. The reasons of conversion included bleeding (15 patients), extensive adhesions in the upper peritoneal cavity (9 patients), high body mass index (BMI; 4 patients with BMI > 30), injury of adjacent organs (3 patients) and mechanical problems (4 patients). The average numbers of retrieved LNs were 30.4 ± 13.4 in the LAG group and 28.1 ± 17.2 in the OG group. There was no obvious difference between the 2 groups (*P* = 0.43). When the retrieved LNs were stratified by the extent of lymph node dissection, there was still no significant difference between the 2 groups. Table 3 summarizes the intra- and post-operative outcomes. The mean surgical time for LAG was 257.8 ± 75.6 minutes, which was longer than that in the OG group (231.0 ± 64.5 minutes). The mean estimated blood loss was 154.5 ± 102.6 mL in the LAG group, which was significantly less than that in the OG group (311.2 ± 118.9 mL, *P* < 0.001). Therefore, fewer transfusions were needed in the LAG group. The postoperative time to passage of flatus was 3.3 ± 1.4 days and 3.9 ± 1.5 days in the LAG and OG groups, respectively. The postoperative time to the initiation of oral intake was 3.7 ± 1.1 days and 4.5 ± 2.0 days in the LAG and OG groups, respectively. Hospital stay after operation was 7.6 ± 2.5 days in the laparoscopic group, which was significantly less than that in the open group (10.7 ± 3.6 days, *P* < 0.001). The intra-operative and postoperative morbidity rate was 6.4% in the LAG group, which was significantly less than that in the OG group (10.5%) (*P* = 0.01). However, there was no significant difference in intra-operative complications between the 2 groups (*P* = 0.25). The incision infection rate in the LAG (6, 1.0%) was less than that in the OG group (15, 2.6%) (*P* = 0.03), and bowel obstruction in the OG group (10, 1.7%) was more common in comparison to the LAG group (3, 0.5%) (*P* = 0.04) (Table 4).

**Table 2:**
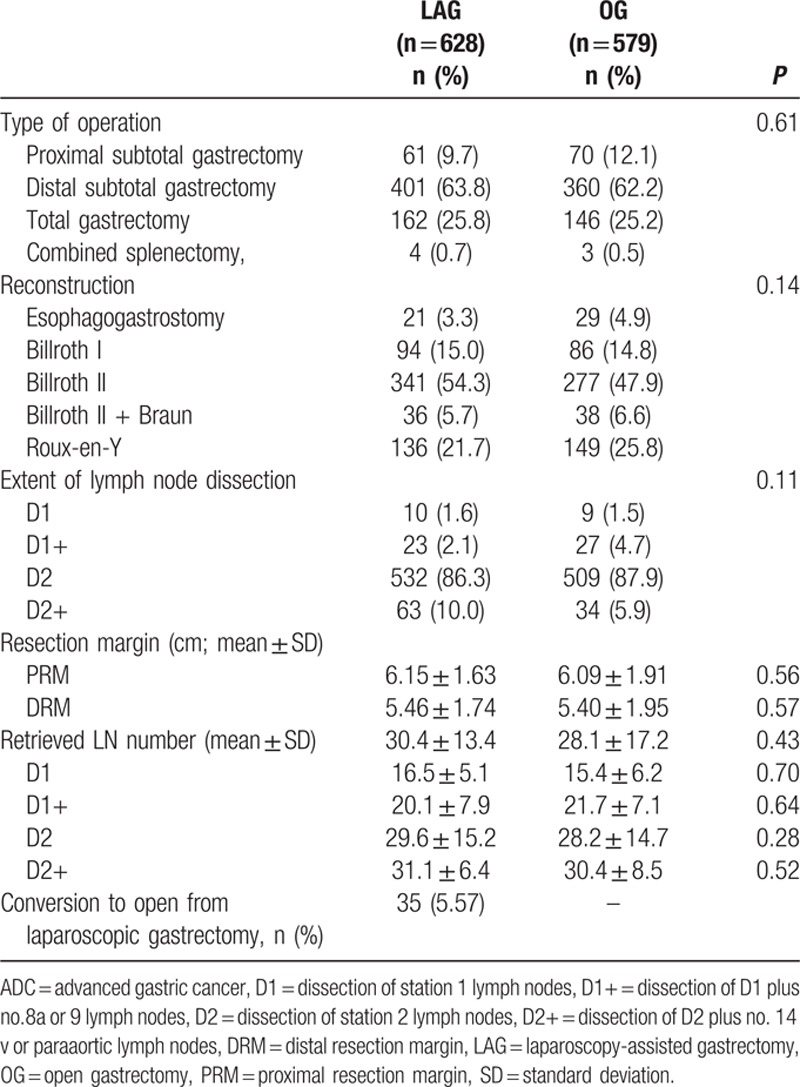
Outcomes of the LAG and OG procedures for AGC.

**Table 3:**
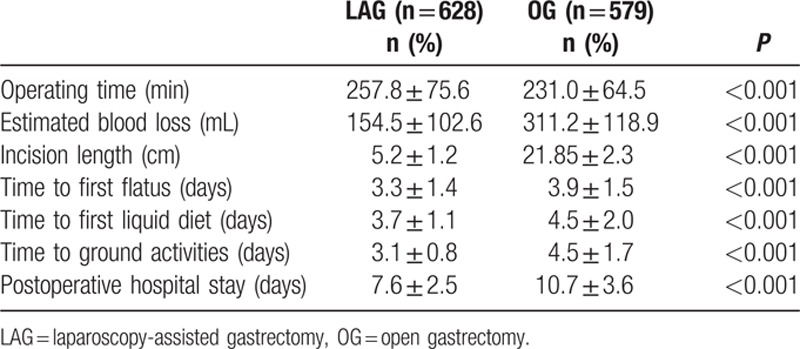
Short-term outcomes of peri-operation in the LAG and OG groups.

**Table 4:**
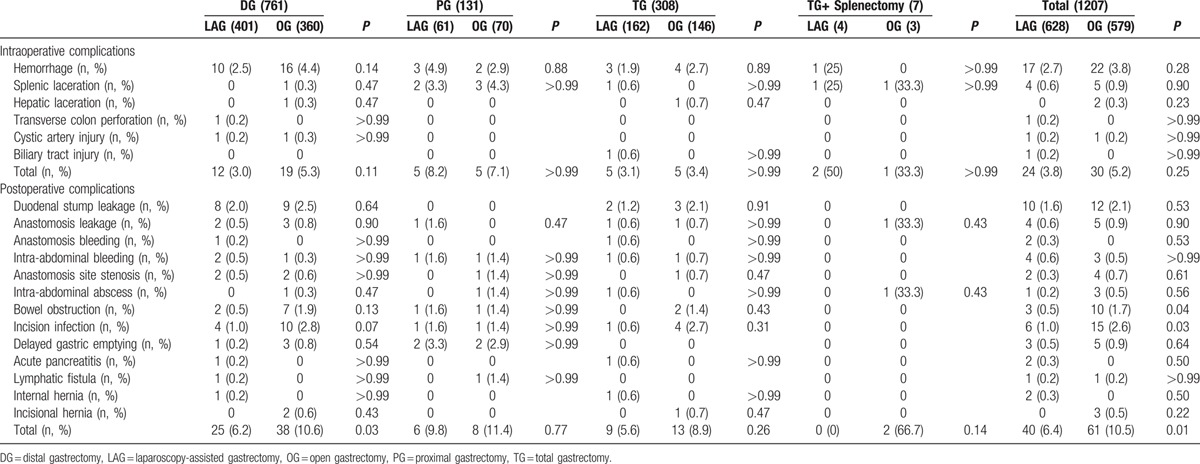
Comparison of intra- and postoperative complication rate between the LAG and OG groups after stratifying by operation type.

### 3.3 Long-term results

**Figure 3:**
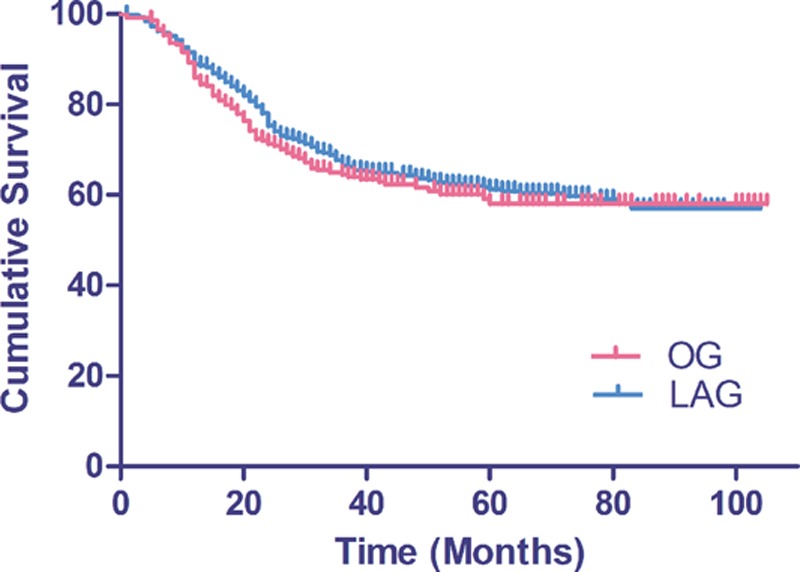
Comparison of the 5-year (overall survival) OS curve of advanced gastric cancer (AGC) patients undergoing laparoscopy-assisted gastrectomy (LAG) and open gastrectomy (OG). The 2 groups did not differ significantly (57.65 vs 53.69%; *P* = 0.22). AGC = advanced gastric cancer, OG = open gastrectomy, OS = overall survival.

**Figure 4:**
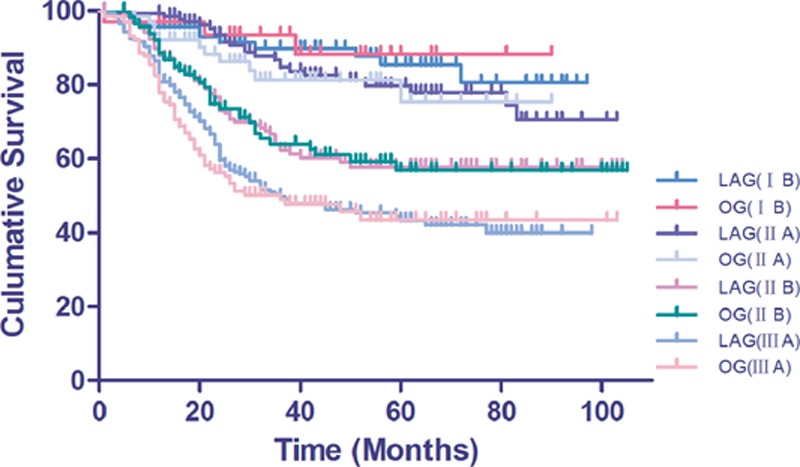
Cumulative curves for overall survival (OS) between the LAG and OG groups according to the TNM stage (AJCC, ver. 7). Overall 5-year survival rates of stage Ib, IIa, IIb, and IIIa were 85.38, 79.70, 57.81, and 34.60% and 88.31, 75.49, 56.84, and 33.08% in the LAG and OG groups, respectively. AJCC = American Joint Committee on Cancer, LGA = left gastric artery, OG = open gastrectomy, OS = overall survival.

Twenty-five patients in the laparoscopic group and 23 in the open group were lost after a median follow-up time of 53.5 months. A total of 221 and 204 patients died of gastric cancer in the LAG and OG groups, respectively. The overall 5-year survival rates were 57.65 and 53.69% in the LAG and OG groups, respectively (Fig. 3). There was no statistically significant difference in the OS rate between groups (*P* = 0.22). The overall 5-year survival rates according to the subclassification for the TNM stage of patients were 85.38%, 79.70%, 57.81%, 34.60% and 88.31%, 75.49%, 56.84%, 33.08% in patients with stage Ib, IIa, IIb, and IIIa, respectively, in both groups. The details are shown in Fig. 4. There were no statistically significant differences in the OS rate for patients with the same TNM stage in the LAG and OG groups. Mortality primarily occurred because of tumor recurrence and distal metastasis. We detected 196 patients (31.2%) with tumor recurrence in the LAG group and 158 patients (27.3%) in the OG group; there was no difference in tumor recurrence between the 2 groups (*P* = 0.14). Recurrence patterns were compared after stratifying by pTNM stages. There were no differences in recurrence patterns between the 2 groups after we stratified them by pTNM stages (Table 5).

**Table 5:**
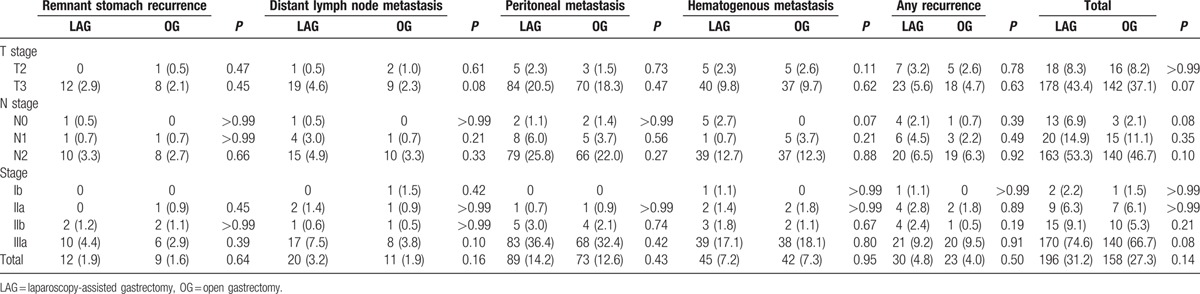
Comparison of the incidence of recurrence and metastasis between the LAG and OG groups after stratifying by pTNM stage.

## 4 Discussion

Kitano et al first performed LAG for early gastric cancer in 1994.^[1]^ Since then, several specialized centers have used LAG for the treatment of EGC.^[2,20,21]^ Some RCT studies of laparoscopic surgery for EGC demonstrated that long-term results were similar or even higher compared to open surgery,^[3–6]^ and LAG has gradually become an alternative standard surgical method for EGC in Japan. However, the application of LAG for AGC remains debatable on account of the difficult technology for performing D2 lymphadenectomy. One retrospective case control study and 1 RCT study that included AGC proved no significant difference in the number of achieved LNs, recurrent rate, or overall survival rate between the 2 groups.^[22,23]^ However, the number of cases was not large enough to prove the oncological safety of LAG for AGC.

The present study included a relatively large number of cases (628 patients in the LAG group and 579 patients in the OG group) who underwent laparoscopy-assisted radical gastrectomies in a single minimally invasive surgery center. The surgeons who performed the operations remained consistent throughout the study, and a specialist was assigned to count the number of retrieved LNs in all gastric cancer patients. We performed LAG safely within an acceptable range of operating times, consistent with previous reports. Because the number of retrieved LNs is the objective index of lymphadenectomy, we compared this factor between the 2 groups. No difference in the number of harvested LNs was found between the 2 groups (30.6 ± 10.1 and 30.3 ± 8.6 in the LAG and OG groups, respectively), which is similar to the results of Chen et al.^[24]^ When the number of retrieved LNs was compared after stratifying by extent of lymph node dissection, there was still no obvious difference between the 2 groups. The number of dissected LNs was sufficient for adequate stage classification in most patients who were treated using D2 LN dissection. The National Comprehensive Cancer Network (NCCN) guidelines for gastric cancer (ver. 1. 2011)^[25]^ suggest no less than the dissection of 15 LNs for radical gastrectomy. The optimal extent of lymphadenectomy in the treatment of gastric cancer continues to be a subject of database. Randle et al^[26]^ reported lymphadenectomy outcomes following D1 or D2 procedure. From 2000 to 2012, 266 (36.6%) which were performed by D1 lymphadenectomy and 461 (63.4%) patients received D2 lymphadenectomy. Recurrence rates were 25.8% and 27.0%, respectively (*P* = 0.74) in D1 and D2 lymphadenectomies. Verlato et al^[27]^ evaluated lymphadenectomy for gastric cancer between Eastern Asia and Western countries by evidence-based medicine. They analyzed the optimal extension of lymphadenectomy in gastric cancer by the surgical guidelines and present literatures. From 2012 to 2013, 2 meta-analyses reported that D2 improves prognosis compared with D1. Now the D2 has been acknowledged as the standard procedure for AGC by the European Society of Surgical Oncology (ESSO), the European Society for Medical Oncology (ESMO), and the European Society of Radiotherapy and Oncology (ESTRO) guidelines. The mean distance of the proximal resection margin was 6.15 ± 1.63 cm in the present study and 5.46 ± 1.74 cm in the distal resection margin in the laparoscopic group. This shows that laparoscopically assisted surgery can achieve an adequate distance from tumor.

The morbidity and mortality rates are important factor for indicating the safety and feasibility of an operation. Different operation types have different incidences of intra- and postoperative complications. So we compared the complication rate between the LAG and OG groups after stratifying by different operation types. The overall surgical morbidity, including intra- and post-operative complications, was 10.2% in the LAG group, which was less than that in the OG group (15.7%). The Korean Laparo-endoscopic Gastrointestinal Surgery Study (KLASS) trial,^[7]^ which was a Korean multicenter prospective randomized study that compared LAG and OG. It reported an 15.1% for the OG group and 11.6% early morbidity for the LAG group, which is consistent with our report. Huscher et al^[22]^ reported LAG-associated morbidity and mortality rates of 26.7% and 3.3%, respectively, and these rates were the same as for open group. The intra-operative complication rate in our study was not significantly different between the 2 groups (*P* = 0.25). However, postoperative complications were more common in the OG group (61, 10.5%) compared to the LAG group (40, 6.4%) (*P* = 0.01), especially in the LADG and ODG groups (6.2%, 10.6%) (*P* = 0.03). The most frequent complications in the OG group were bowel obstruction and incision infection. The reasons for these differences may be due to the longer incision length in the OG group (21.85 ± 2.3 cm) compared to the LAG group (5.2 ± 1.2 cm). However, the other complications were not clearly different between the 2 groups, such as duodenal stump leakage and bleeding, intra-abdominal bleeding and abscess. Mochiki et al^[28]^ and Lee et al^[29]^ reported that the common complications after LAG were anastomosis leakage and anastomosis site stenosis. These differences may have occurred because we routinely sutured the anastomosis and duodenal stump after the application of Endo-GIA or the endoscopic endonasal approach (EEA), and repeat endoscopy was performed to visualize anastomosis hemorrhage or leak.

Conversion to open procedure was 35 cases (5.57%) because of intraoperative complications in our study. Bleeding was the most reason to convert to open surgery, primarily resulting from injury to the spleen, right gastric artery, or short gastric vessels, which are adjacent to the upper of the spleen. Complications intra-operation occurred more frequently during LATG than other laparoscopic procedures. These results are similar to those of Lee et al,^[30]^ who reported a 5.2% rate of conversion to open surgery in LAG. We also found that the rate of conversion to open surgery in the first 3 years of the study period (20/167, 12.0%) was greater than in the later years (2007 to 2011, 15/461, 3.3%). Kim^[31]^ compared the overall postoperative complication rate before and after learning curve completion and demonstrated a significant decrease after learning curve completion (43.3% vs 19.0%; *P* < .01). Kunisaki et al^[32]^ reported a comparative study of LADG and open distal gastrectomy (ODG) in Japan. Operation time and postoperative hospital stay were not significantly different in the LADG group from the ODG group after 60 LADG cases. These studies suggest that 50 to 60 cases are required to complete the LADG learning curve. Therefore, the patient's condition, the surgeon's laparoscopic skill, and the stability of the surgical team affect the conversion and intra-operative complication rates. It is necessary to operate with the help of an experienced laparoscopic surgeon during the learning-curve period.^[33]^

Although oncologic outcome after laparoscopic versus open gastrectomy for treatment of advanced gastric cancer has been reported in some studies.^[8,10,22]^ The number of patients was relatively small and some series analyzed mainly early gastric cancer. The present study followed-up 628 patients in the LAG group and 579 patients in the OG group, and there was 228 (36.3%) and 210 (36.2%) IIIa stage patients in the 2 groups, respectively. The median follow-up period was 53.5 months. The 5-year OS rates were not different between the LAG and OG groups (57.65 and 53.69%, respectively). These survival rates are lower than those in the study by Shinohara et al, which reported rates of 68.1% for LAG and 63.7% for OG.^[34]^ These results may have occurred because a large pool of stage IIIa patients (228/36.3%) was examined in our study compared to 48 (25.8%) in the Shinohara study. Comparisons of same-stage patient survival curves in the LAG and OG groups revealed no differences in the 5-year survival rates among same-stage patients between the 2 groups. These results demonstrated that the surgical method did not affect long-term survival rates in AGC patients staged below T3.

The recurrence rates in our study (31.2% in LAG and 27.3% in OG) were no significant difference (*P* = .14) between the 2 groups, even it was stratified by the pT and pN stages. However, the recurrence rate in our study was higher compared to previous reports. Kim et al^[35]^ reported 25 patients (3.3%) with tumor recurrence, including 1.4% (8/592) in EGC and 10.6% (17/161) in AGC. Song et al^[36]^ analyzed the recurrence rates of LAG for gastric cancer and demonstrated that the incidence of recurrence was 3.5% in all patients, 1.6% in EGC, and 13.4% in AGC. The primary reason for these differences is likely that our study included more stage IIIa patients than the Kim and Song studies. The recurrence sites in our study included the peritoneum, liver, LN metastasis, and remnant stomach, which is similar to the findings of Lee^[14]^ and Song et al.^[36]^ Peritoneal spread is the major route of gastric cancer metastasis. Our previous study for gastric cancer demonstrated that laparoscopic surgery has less of an impact than conventional surgery on inflammatory factors implicated in local recurrence and peritoneal metastasis because of its decreased impact on postoperative immune responses, both in the peritoneum and systemically.^[37]^ We observed that the recurrence patterns in each stage were similar to conventional open surgery, which indicates that the laparoscopic procedure did not increase the rates of local and peritoneal recurrence, even in N+ or T3 stage. Recent studies have also reported no increase in the rate of peritoneal recurrence after LAG.^[8,38,39]^ One early study of LAG performance reported trocar metastasis in some patients.^[40]^ However, only 2 patients in our study exhibited trocar metastasis in the first year, and no trocar metastasis occurred in subsequent years after we applied a lower (10–12 mm Hg) CO_2_ pneumoperitoneum pressure and decreased the pressure before the trocars were removed. Our previous research demonstrated that LAG did not enhance the risk of free gastric cancer cell detection rates compared with OG.^[41]^ Basic research in vitro also demonstrated that CO_2_ pneumoperitoneum did not increase the proliferation rate and invading ability of gastric cancer cells.^[42,43]^

In order to enhance the 5-year survival rate of advanced gastric cancer, some centers started neoadjuvant chemotherapy from 1996. In the last 20 years, large-scale randomized trials have demonstrated the efficacy of neoadjuvant chemotherapy: including adjuvant chemoradiation treatment (INT-0116 trial),^[44]^ adjuvant single-drug chemotherapy (ACTS-GC trial),^[45]^ and perioperative 3-drug combination chemotherapy (MAGIC trial).^[46]^ Adjuvant and neoadjuvant chemotherapies are now increasingly used with surgery for locally AGC. There are several advantages of a neoadjuvant approach for treating local advanced disease. Which were as follows: treating the distant microscopic disease early, the ability to evaluate the response of therapy, and downstaging of tumor to enhance resectability. ACTS-GC trial^[45]^ and CLASSIC trial^[47,48]^ demonstrated superior overall survival (OS) with neoadjuvant perioperative chemotherapy compared with surgery alone. Both United States and European guidelines^[49,50]^ consider preoperative chemotherapy as the preferred pathway for ≥ T2 and/or N ± gastric cancer reaching the “level 1” of recommendation in the National Comprehensive Cancer Network Consensus. However, there are some disadvantages in neoadiuvant chemotherapy for GC, including toxic reactions, tissue edema and even no working to tumor. So the neoadjuvant therapy was still considered investigational by the last edition of the Japanese Gastric Cancer Association guidelines while researchers await the results of dedicated ongoing trials.^[17]^ Although we started neoadjuvant treatment from 2009 (these data did not show in this paper), we found that neoadjuvant treatment could enhance the difficulty of operating. So we believed that such factors must be taken into account in order to develop a personalized treatment plan.

In conclusion, LAG with radical LN dissection is a feasible and safe procedure in patients with no more than T3 stage AGC. LAG for AGC should be performed under strict indications, which include less than T3 stage, negative peritoneal cavity liquid cytology and no distal metastasis. The application of LAG for AGC must follow the same oncological principles as the traditional open procedure. However, this research was a nonrandomized single-center study with limitations; therefore, a large-scale prospective RCT study is necessary for LAG to be accepted as an alternative or standard treatment for AGC.
